# Supplementary material for: The Influence of Home-Based Music Therapy Interventions on Relationship Quality in Couples Living with Dementia—An Adapted Convergent Mixed Methods Study
Source: Int J Environ Res Public Health. 2023 Feb 6;20(4):2863. doi: 10.3390/ijerph20042863 (PMC9966062; doi:10.3390/ijerph20042863)
Supplement: Supplementary file 1 [file ijerph-20-02863-s001.zip › Interveiw guide ENGLISH baseline and post.docx.pdf]

Music, dementia and relationship project
Interview guide – Interview baseline

Please note: This is a translation of the original interview guide, which was in Norwegian

The interview is done with the couple together. Both parties are also given the opportunity to have a conversation alone if they wish, in connection with the joint interview. This is offered as an opportunity for both to elaborate, or provide additional information for the joint interview. The same interview guide is then used as a starting point.

Attention before starting the interview: has consent been obtained from both parties? Have you recently assessed consent competence?

Before the interview: introduce yourself again. Thank you so much for giving their time to be part of this, it is of great importance for the research and can benefit many people in the long run.

Introduction:
I'm interested in getting to know you as a couple, and want you to share as much about yourselves as you feel comfortable. I will record the interview with a tape recorder, and only I and my supervisors will be able to hear the recording. The recording is deleted as soon as it has been printed out as text, and all identifying information that may appear in the interview is anonymised. You will have the opportunity to read through the printed interview before it is used in the research. You have given written consent to participate in this. I would like to remind you that at any time, and without giving any reason, you can refrain from answering questions I ask, ask me to turn off the tape recorder, ask for a break or for us to end the interview altogether. We can get into topics that are personal, and then this is extra important for you to know about.

I want to ask questions about the two of you as a couple, questions about your relationship with music, and about how the dementia diagnosis affects your life.

Questions:

We start with the two of you as a couple:

- Would you like to tell me a little about you as a couple?

Probes:

- How did you meet, for example?
- Important events in common life?
- Children?
- Residences?
- Work and leisure?
- Hobbies, interests, activities?
- Challenges?
- Good days?
- Your relationship: closeness, cooperation, friendship, romance, intimacy?
- How would you describe [name]? (Ask both)
- How are you two together at your best? Can you describe a situation, event?

Now I want to know a little about their relationship with music:

- In what way has music been part of your life as a couple?

Probes:

- Concerts?
- Listening to music at home?
- Play sing yourself?
- Children and leisure activities?
- Friends?
- Together or individually?
- What do you think about doing music together at home in this project?

About the couple, the dementia:

Do you feel that the relationship has changed after the dementia diagnosis (or equivalent)?

Probes:
- Practical, chores, activities
- Communication, how you talk together, how you solve challenges?
- Feelings?
- Closeness and intimacy?
- Were there things you did before that you no longer do?
- Are there any things you have started doing that you didn't do before?

Finally: Is there anything you would like to say, tell me about or ask me about before we end?

Thank you very much for this conversation! We will be together several times in the future, but remember that you can also contact me or my main supervisor Tone Kvamme at any time if there is anything you are wondering about or want to say.
You can find the contact information in the information letter.
Now I turn off the tape recorder.
Thanks again for now

Music, dementia and relationship project
Interview guide – Interview post intervention

Please note: This is a translation of the original interview guide, which was in Norwegian

The interview is done with the couple together. Both parties are also given the opportunity to have a conversation alone if they wish, in connection with the joint interview. This is offered as an opportunity for both to elaborate, or provide additional information for the joint interview. The same interview guide is then used as a starting point.

Attention before starting the interview: has consent been obtained from both parties? Have you recently assessed consent competence?

Before the interview: introduce yourself again. It's been a few weeks since we last saw each other, and I can't wait to talk to you again! Thank you for giving up your time to take part in this, it is of great importance for the research and can benefit many people in the long run.

Introduction:

I'm interested in hearing about how you're doing now, and what it's been like doing music together. I would also like to ask if it is the case that the music influences your feelings as a couple. I want you to tell as much about yourself as you feel comfortable. I will record the interview with a tape recorder, and only I and my supervisors will be able to hear the recording. The recording is deleted as soon as it has been printed out as text, and all identifying information that may appear in the interview is anonymised. You will have the opportunity to read through the printed interview before it is used in the research.

You have given written consent to participate in this. I would like to remind you that at any time, and without giving any reason, you can refrain from answering questions I ask, ask me to turn off the tape recorder, ask for a break or for us to end the interview altogether. We can get into topics that are personal, and then this is extra important for you to know about.

Questions:

It's been a while since we've seen each other. I would therefore first like to hear from you how you are doing? Is there anything you would like to say, tell me about or ask me about, before I start with my questions?

How have the last 14 weeks been for you?

Has something happened that you want to tell about?

- Nice or less nice?

Have you done anything together that was enjoyable or funny?

- Can you tell me more about it?

Have you ever experienced something that was difficult or embarrassing?

- Can you tell me more about it?

I now want to ask questions about whether you have been involved in music together, and then about the two of you as a couple.

First, music:

Have you been making music at home together these weeks?

Probes:

- What?
- How often?

- How have you experienced this?

Probes:

- Is there anything about the music that has been particularly nice/pleasant/funny/good?
- Is there anything about the music that has been particularly demanding/challenging/difficult?

- How do you feel about doing music with [name]? (Ask both)

Probes:

- Do you think that music can help in any way? How?
- Do you think that music can have something to say about how you see each other? How?
- Do you think that music can have something to say about collaboration? How?
- Do you think that music can have something to say about the closeness between you? How?

How are you together as a couple now?

Probes:

- Do you experience good days and moments?
- Are you experiencing challenges?
- Your relationship during this period: closeness, cooperation, friendship, romance, intimacy?
- Practical, chores, activities?
- Communication, how you talk together, how you solve challenges?
- Were there things you did before that you no longer do?
- Are there any things you have started doing that you didn't do before?

- How would you describe [name]? (Set both)
- How are you two together at your best? Can you describe a situation, event?

Finally: Is there anything you would like to say, tell me about or ask me about before we end?

Thank you very much for this conversation, and for the cooperation! This was our last planned meeting. It has been a great pleasure to get to know you! You can still contact me or my main supervisor Tone Kvamme if there is anything you are wondering about or want to say. You can find the contact information in the information letter. I will also update you by e-mail or phone about what happens next in the project, if you wish.
Now I turn off the tape recorder.
Thanks again for now.
