# Supplementary material for: The Influence of Home-Based Music Therapy Interventions on Relationship Quality in Couples Living with Dementia—An Adapted Convergent Mixed Methods Study
Source: Int J Environ Res Public Health. 2023 Feb 6;20(4):2863. doi: 10.3390/ijerph20042863 (PMC9966062; doi:10.3390/ijerph20042863)
Supplement: Supplementary file 1 [file ijerph-20-02863-s001.zip › QCPR.pdf]

QCPR - Quality of Caregiver and Patient Relationship: 14-item-version

(Spruytte, Van Audenhove & Lammertyn, 2000)

Answering categories:
1 = totally disagree
2 = disagree
3 = undecided
4 = agree
5 = totally agree

1. We often spend time together in a pleasant way
2. We often disagree
3. A great distance exists between us
4. We accept each other the way we are
5. When problems occur, we mostly can solve these problems easily
7. I get along well with [him/her]
10. We offer each other tenderness
11. I often get irritated by [him/her]
13. When I am with [him/her], I feel very well
14. We often try to impose each other's opinion
17. I blame [him/her] for being the cause of my problems
19. We appreciate each other as a person
21. [He/she] does not appreciate enough everything I do for him/her
22. When I haven't seen [him/her] for a while, I am happy to see him/her again

Positive subscale on warmth and affection: items 1, 4, 5, 7, 10, 13, 19, 22

Negative subscale on conflict and criticism: items 2, 3, 11, 14, 17, 21
These items are scored reversed.
